# Effects of exercise intensity on white adipose tissue browning and its regulatory signals in mice

**DOI:** 10.14814/phy2.15205

**Published:** 2022-03-14

**Authors:** Riku Tanimura, Leo Kobayashi, Takanaga Shirai, Tohru Takemasa

**Affiliations:** ^1^ Graduate School of Comprehensive Human Sciences University of Tsukuba Tsukuba Japan; ^2^ JIJI PRESS Ltd Cyuo‐ku Japan; ^3^ Research Fellow of the Japan Society for the Promotion of Science Tokyo Japan; ^4^ Faculty of Health and Sports Sciences University of Tsukuba Tsukuba Japan

**Keywords:** adipose tissue, browning, exercise, myokine

## Abstract

Adipose tissue has been classified into white adipose tissue (WAT), brown adipose tissue (BAT), and beige adipose tissue the latter of which is produced as WAT changes into BAT due to exposure to cold temperature or exercise. In response to these stimulations, WAT produces heat by increasing mitochondrial contents and the expression of uncoupling protein 1 (UCP1), thus facilitating browning. Exercise is known to be one of the triggers for WAT browning, but the effects of exercise intensity on the browning of WAT remain to be unclear. Therefore, in this study, we aimed to examine the effects of high‐ or low‐intensity exercises on the browning of WAT. Mice performed high‐ or low‐intensity running on a treadmill running 3 days a week for four weeks. As per our findings, it was determined that four weeks of running did not significantly reduce inguinal WAT (iWAT) wet weight but did significantly reduce adipocytes size, regardless of exercise intensity. The protein expression level of UCP1 was significantly increased in iWAT by high‐intensity running. In addition, the expression of oxidative phosphorylation proteins (OXPHOS) in iWAT was significantly increased by high‐intensity running. These results demonstrated that high‐intensity exercise might be effective for increasing mitochondrial contents and heat production capacity in iWAT. Furthermore, we found that high‐intensity running increased the protein expression level of fibroblast growth factor 21 (FGF21) in skeletal muscle compared with that in low intensity running. We have also examined the relationship between browning of WAT and the expression of FGF21 in skeletal muscle and found a positive correlation between the protein expression of UCP1 in iWAT and the protein expression of FGF21 in gastrocnemius muscle. In conclusion, we suggest that high‐intensity exercise is effective for the browning of WAT and the increase of FGF21 in skeletal muscle.

## Graphical abstract

We aimed to examine the effects of high‐ or low‐intensity exercises on the browning of WAT. Mice performed high‐ or low‐intensity running on a treadmill running 3 days a week for four weeks. As a result, the protein expression level of UCP1 was significantly increased in iWAT by high‐intensity running.

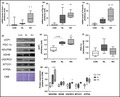

## 1 INTRODUCTION

Adipose tissue can be classified into two types, according to their function: white adipose tissue (WAT) and brown adipose tissue (BAT) (Kurylowicz & Puzianowska‐Kuznicka, 2020). WAT is the adipose tissue that constructs visceral and subcutaneous fat and stores excess energy in the body as triglycerides. Obesity is known to be caused by excessive accumulation of WAT (Vishvanath & Gupta, 2019). Conversely, BAT located at the interscapular region of humans and rodents, it is known to contain many mitochondria in its cells and express high levels of the uncoupling protein 1 (UCP1). This protein is related to the dissipation of energy through heat production. Based on these features, it is suggested that BAT is involved in the maintenance of body temperature homeostasis regulation of energy balance and body weight (BW), and metabolism of glucose and lipids (Rui, 2017). Meanwhile, beige adipose tissue is known as the third type of adipose tissue (Ishibashi & Seale, 2010). Similar to BAT, beige adipose tissue is known for its ability to produce heat due to the expression of UCP1 and is rich in mitochondria (Shabalina et al., 2013). Although beige adipose tissue has these similar characteristics to BAT, its development is different (Sidossis & Kajimura, 2015). BAT is derived from the Myf5^+^ myogenic lineage (Seale et al., 2008), while beige adipose tissue is derived from the Myf5^−^ adipose progenitor cell or some populations of WAT (Wang et al., 2013; Wu, Bostrom, et al., 2012). The process of WAT browning involves the expression of transcription factors, such as the PR domain‐containing 16 (PRDM16), peroxisome proliferator‐activated receptor‐γ (PPAR‐γ), and UCP1; which is the hallmark of thermogenesis (Shabalina et al., 2013). Moreover, browning of WAT increases its mitochondrial content and capillary vessels, both of which aid in the enhancement of energy metabolism.

Browning of WAT is often caused by exercise (Dewal & Stanford, 2019; Martin et al., 2020; McKie & Wright, 2020). Exercise induces metabolic adaptations, such as the enhancement of glucose metabolism through activation of AMP‐activated protein kinase (AMPK) and oxidative metabolism by an increase or activation of mitochondria in skeletal muscle (Hoshino et al., 2016). Previous studies have reported that exercise intensity is one of the factors that can alter the molecular changes in skeletal muscle and blood components (Fiorenza et al., 2018; MacInnis & Gibala, 2017; Takeda & Takemasa, 2015). However, exercise induces metabolic alterations in adipose tissue as well as skeletal muscle (Liepinsh et al., 2020). Previous studies on mice indicated that chronic treadmill running induces an increase in the protein expression of UCP1 of in subcutaneous WAT (Trevellin et al., 2014). Voluntary running decreased the mass of WAT in obese mice (Bell et al., 1995). In short, exercise induces the reduction of fat mass and molecular adaptation of thermogenesis in WAT.

The effect of myokines has been suggested as one of the factors that induce browning of WAT by exercise (Graf & Ferrari, 2019). A recent study reported that these hormone‐like peptides, secreted from skeletal muscle, induce adaptations in the body metabolism (Furuichi et al., 2018; Pedersen & Febbraio, 2008). Furthermore, myokines have been reported to have regulatory effects on metabolism in skeletal muscles, in addition to a variety of tissues including adipose tissue, liver, pancreas, bone, and the brain (Barbalho et al., 2020). Exercise induces the secretion of myokines such as fibronectin type III domain‐containing protein 5 (FNDC5)/irisin, fibroblast growth factor 21 (FGF21), and interleukin‐6 (IL‐6) (Bostrom et al., 2012; Lee et al., 2014). These myokines have been reported to cause the browning of WAT (Adams et al., 2012; Cho et al., 2021).

A number of studies have focused on the browning of WAT during exercise, but how exercise prescription potentially is effective for recruiting beige adipocytes remains undetermined. Studies focused on exercise intensity, have concluded that higher intensity exercise is more effective to improving high‐fat diet‐induced adiposity, inflammation, and glucolipid metabolic disorders, and in promoting the phenotypic switch from white to brown adipose tissue (Wang et al., 2017). However, the mechanism by which myokines stimulate the intensity‐dependent browning of WAT is yet to be elucidated. Previous studies have reported that a higher intensity endurance exercise induced higher response of FGF21 in humans (He et al., 2019). Moreover, the protein expression of FNDC5 in skeletal muscle was increased by high intensity exercise above lactate threshold (LT), but not in moderate intensity exercise in rat (Constans et al., 2021). Based on these reports, we hypothesized that the intensity‐dependent expression of myokines involved in browning would be observed in mice. Therefore, we aimed to examine the effects of high‐ and low‐intensity exercise, those were based on LT on the browning WAT and the expression of myokines; FGF21 and FNDC5.

## 2 MATERIALS AND METHODS

### 2.1 Experimental animals

Seven‐week‐old male C57BL/6J mice were purchased from Charles River Laboratories Japan, Inc; they were housed at temperature (22 ± 2°C) and humidity (55% ± 5%)‐controlled holding facilities under a 12‐/12‐h light/dark cycle and with *ad libitum* access to food and water. The animals were divided into three groups: sedentary mice (CON, *n* = 5–7), low‐intensity running mice (RL, *n* = 5–7) and high‐intensity running mice (RH, *n* = 5–7).

After four weeks of exercise intervention, the mice were euthanized by cervical dislocation, and the gastrocnemius muscle and inguinal WAT (iWAT) were excised, weighed, quickly frozen in liquid nitrogen and stored at −80°C until further use.

### 2.2 Exercise protocol

**FIGURE 1:**
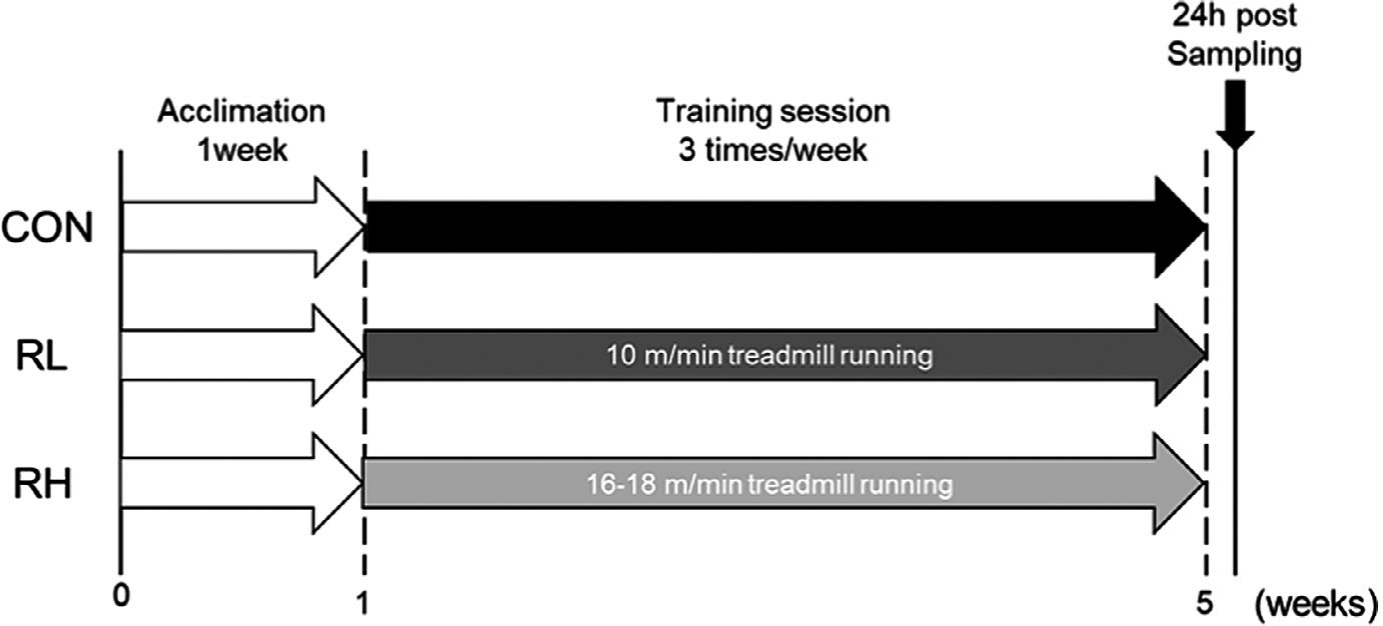
Experimental design. Mice were divided into three groups, that is, sedentary mice (CON), low‐intensity running mice (RL), and high‐intensity running mice (RH), with five to seven mice in each group. After 1‐week acclimation, we performed high‐ or low‐intensity treadmill running 3 days/week for 4 weeks

Running groups of mice were made to run for one hour on a rodent treadmill. The running speed was set at 10 m/min for the RL group, whereas it was 16–18 m/min for the RH group based on previous report (Ferreira et al., 2007). Running groups of mice were exercised three times a week for four weeks. The experimental protocol is shown in Figure 1.

### 2.3 Histological analysis

iWAT adipose tissues were fixed in 10% phosphate‐buffered formalin for 1 day and processed for paraffin sectioning. Tissue sections of 5 μm were cut and stained with hematoxylin and eosin for examination by microscopy. To quantify adipocyte size, the stained sections were measured by tracing the outline of each cell using the Fiji image processing package in the ImageJ Software.

### 2.4 Western blotting

All tissue was frozen immediately in liquid nitrogen, and total protein was extracted in lysis buffer containing 50 mM of HEPES (pH: 7.6), 150 mM NaCl, 10 mM EDTA, 10 mM Na4P2O7, 10 mM NaF, 2 mM Na3VO4, 1% (v/v) NP‐40, 1% (v/v) Na‐deoxycholate, 0.2% (w/v) sodium dodecyl sulfate (SDS), and 1% (v/v) of a complete protease inhibitor cocktail. Protein concentrations were measured using a Protein Assay Bicinchoninate Kit (Nacalai Tesque Inc.). Prior to SDS–polyacrylamide gel electrophoresis (PAGE) analysis, an aliquot of the extracted protein solution was mixed with an equal volume of sample loading buffer containing 1% (v/v) 2‐mercaptoethanol, 4% (w/v) SDS, 125 mM Tris–HCl (pH 6.8), 10% (w/v) sucrose, and 0.01% (w/v) bromophenol blue. Five micrograms of protein were separated by SDS–PAGE and electrically transferred to an Immuno‐Blot polyvinylidene fluoride or polyvinylidene difluoride membrane (Bio‐Rad Laboratories). The blot was blocked using Blocking One (Nacalai Tesque Inc.) for 1 hour at room temperature and incubated with primary antibodies overnight at 4°C in Tris‐buffered saline with 0.1% Tween‐20. Membranes were then incubated with horseradish peroxidase‐conjugated secondary antibody for 60 min at room temperature. Signals were detected using the ImmunoStar Zeta or LD (FUJIFILM Wako Pure Chemical Co), quantified by C‐Digit (LI‐COR Biosciences), and reported as arbitrary units. Coomassie Brilliant Blue staining was used to verify consistent loading.

### 2.5 Primary antibodies for Western blotting

The following primary antibodies were used for Western blotting: anti‐ AMPK (#2532S; Cell signaling technology), anti‐ phospho‐AMPK (#2535S; Cell signaling technology), acetyl‐CoA carboxylase (ACC) (#3676; Cell Signaling Technology), p‐ACC (#3661; Cell Signaling Technology), anti‐ UCP1 (ab10983; Abcam), anti‐PGC‐1α (516557; Merck Millipore), anti‐oxidative phosphorylation (OXPHOS) (ab110413; Abcam), anti‐ FGF21 (sc‐81946; Santa Cruz), and anti‐ FNDC5 (ab174833; Abcam).

### 2.6 RNA isolation and real‐time polymerase chain reaction

Total RNA (mRNA) was isolated from frozen whole gastrocnemius muscles and iWAT using the TRIzol reagent (Invitrogen). The quantity and quality of RNA were validated with NanoDrop (Thermo Fisher Scientific). Complementary DNA was synthesized using the PrimeScript RT Master Mix (Takara Bio, Inc.). qRT‐PCR was performed with the Thermal Cycler Dice Real‐Time System using SYBR Premix Ex Taq II (Takara Bio, Inc.). The PCR protocol was as follows: denaturation for 15 s at 95°C, then annealing and extension for 40 s at 60°C (40 cycles). The dissociation curve for each sample was analyzed to verify the specificity of each reaction. The relative mRNA expression levels of the target genes were determined by the delta‐delta Ct method and normalized to the expression of hypoxanthine phosphoribosyltransferase (Hprt). The primer sequences are shown in Table 1.

**TABLE 1:** Primer sequence for RT‐PCR

Gene	Forward primer (5′‐3′)	Reverse primer (5′‐3′)
*Pgc‐1α*	CCCTGCCATTGTTAAGACC	TGCTGCTGTTCCTGTTTTC
*Prdm16*	AGTCGGACAACCATGCACTT	GTTCTCACAGGCCGTTTGTCCA
*Ucp1*	ACTGCCACACCTCCAGTCATT	CTTTGCCTCACTCACTCAGGATTGG
*Fndc5*	TTGCTCCAGAATGCAGACCG	TAAACGCGGGCAGTACCTTC
*Fgf21*	CTGCTGGGGGTCTACCAAG	CTGCGCCTACCACTGTTCC
*Hprt*	CAGCCCCAAAATGGTTAAGGTT	TCCAACAAAGTCTGGCCTGTAT

### 2.7 Statistical analysis

Data are shown as means + SD. For all measurements, one‐way analysis of variance was conducted. When a significant *p* value was obtained, statistical significance was calculated according to Tukey's method. The SPSS software (IBM Corp.) was used for all statistical calculations and the significance level was set to *p *< 0.05 for all cases. Correlations were analyzed using Pearson's correlation test. The significance level was set to *p *< 0.05.

## 3 RESULTS

### 3.1 Body and tissue weight

**FIGURE 2:**
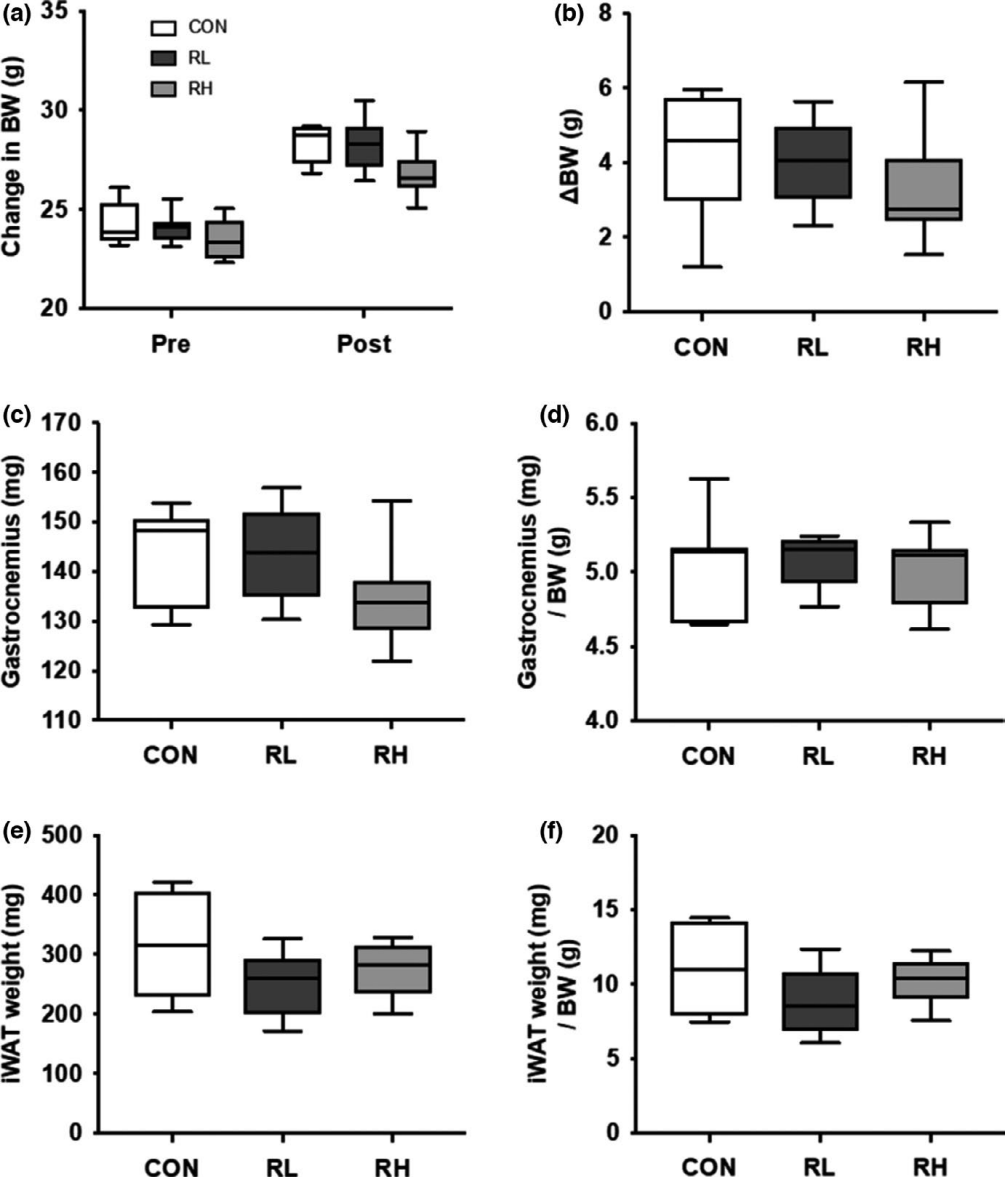
Effect of high‐ or low‐intensity running on BW, gastrocnemius wet weight, and iWAT wet weight. Pre and post training BW (A), change in BW at pre and post training (B), gastrocnemius muscle wet weight (C), gastrocnemius muscle wet weight per BW (D), iWAT wet weight (E) and iWAT wet weight per BW (F), and were measured after 4 weeks of running. CON, control; RL, low intensity running; RH, high intensity running. Data are presented as means + SD

The changes in BW were measurand before (pre) and after (post) 4 weeks training. Wet weight of gastrocnemius muscle and iWAT were measured following four‐weeks exercise training. Here, no significant difference was noted in BW at pre and post training (Figure 2a). There was less of an increase in BW in the training groups, but no significant differences were identified among the groups (Figure 2b). There was also no significant difference in gastrocnemius muscle wet weight and gastrocnemius muscle wet weight/BW (Figure 2c,d). The wet weight of iWAT and iWAT wet weight/BW were noted to be lower in the training groups, but no significant differences were identified between all groups (Figure 2e,f).

### 3.2 iWAT adipocyte size

**FIGURE 3:**
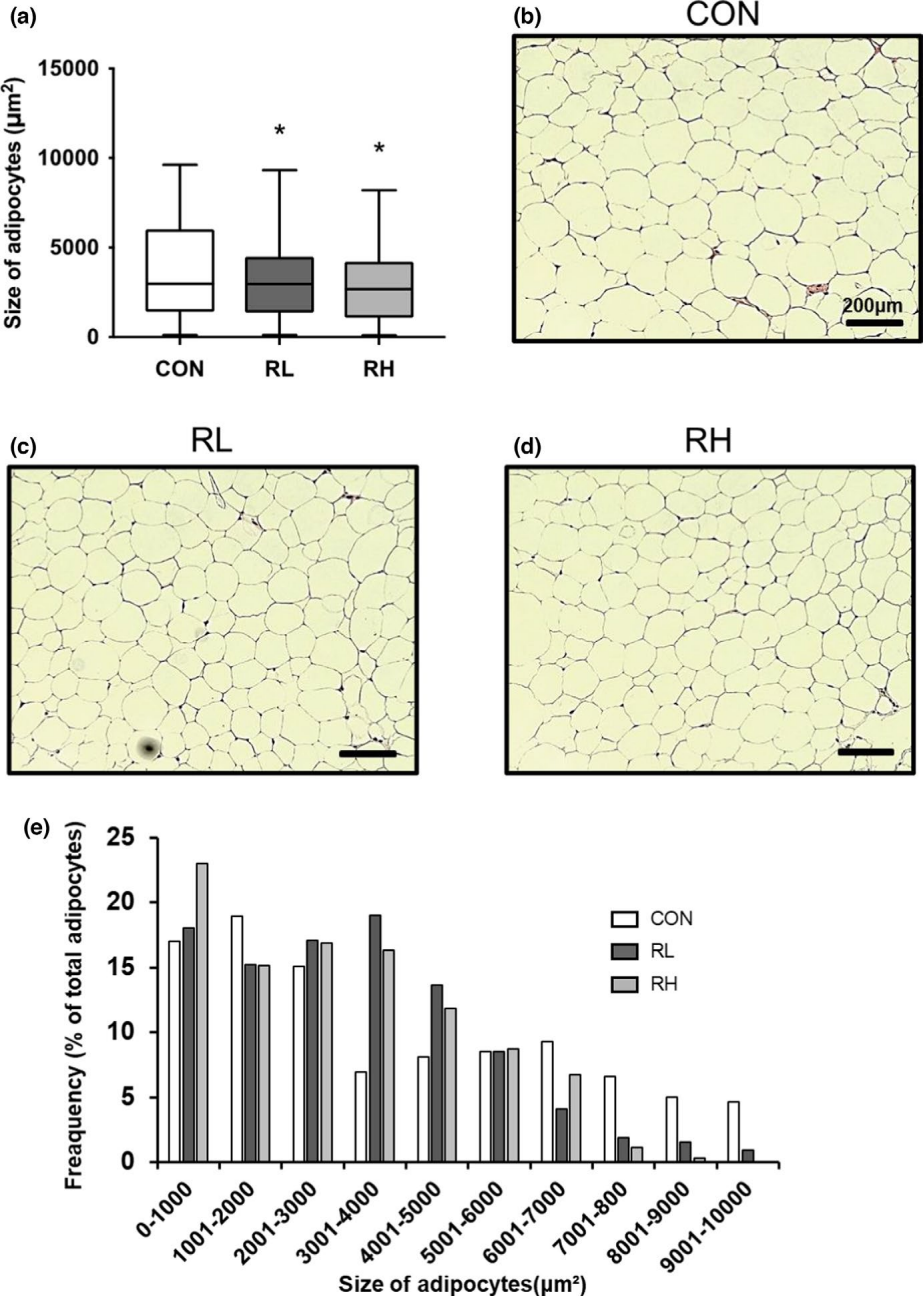
Effect of high‐ or low‐intensity running on iWAT adipocyte size (A). Representative microscopic images of adipocyte in CON (A), RL (B), and RH (C). Distributions of adipocyte size for each experimental group (E). Data are presented as means + SD. *p < 0.05 versus CON+ group

We next investigated the adipocyte reduction effects of high‐ or low‐intensity running in mice. Adipocyte size of the iWAT was significantly decreased in the running groups compared to the control group (Figure 3a–e). These results indicate that running exercise reduced iWAT adipocyte size in mice.

### 3.3 Metabolism and mitochondria related proteins in gastrocnemius muscle

**FIGURE 4:**
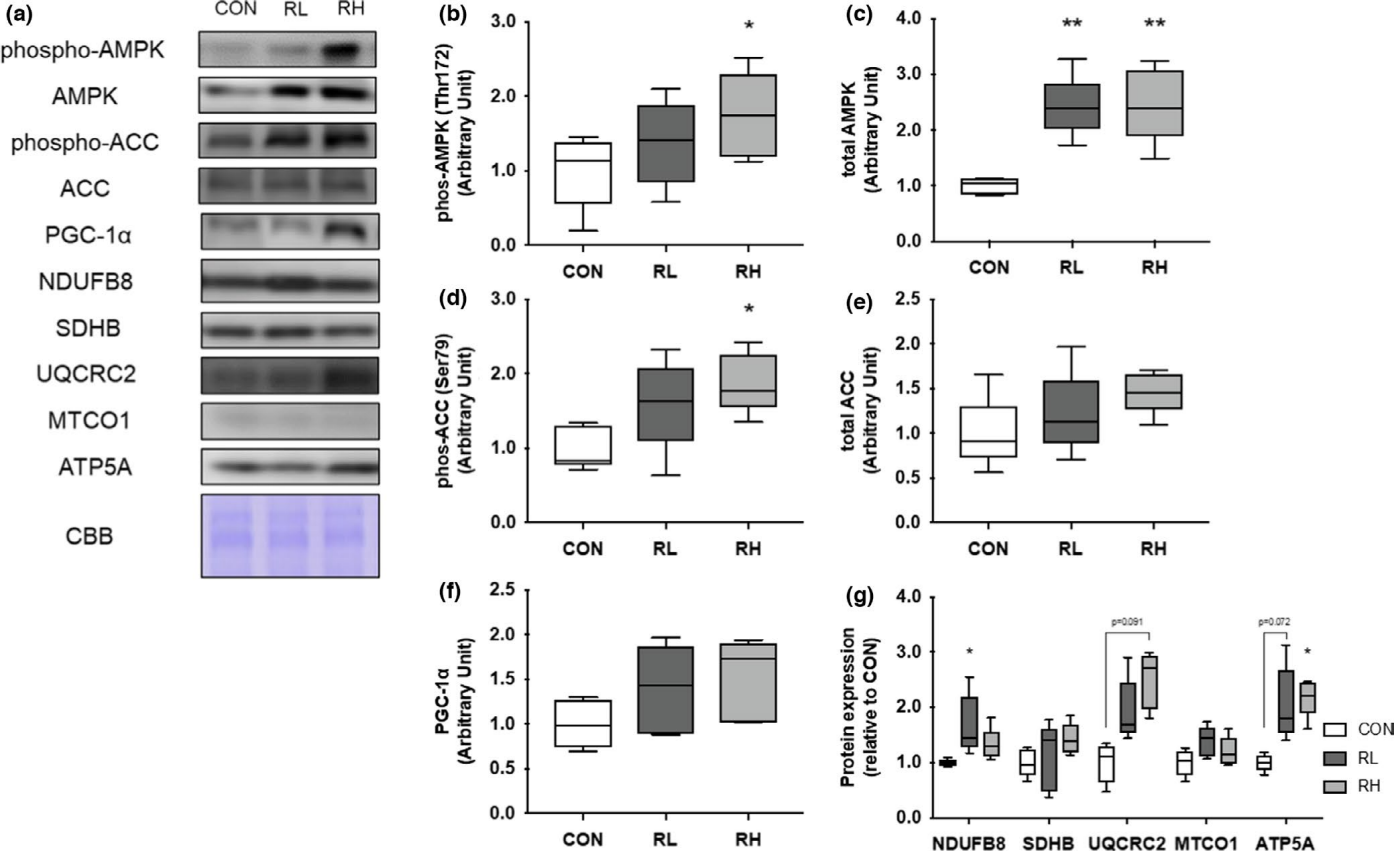
Effect of high‐ or low‐intensity running on oxidative metabolism and mitochondrial related proteins in the gastrocnemius muscle. Representative immunoblots are shown in (A). Phosphorylation of AMPK (B), total expression of AMPK (C), phosphorylation of ACC (D), total expression of ACC (E), expression of PGC‐1α (F), and OXPHOS (G) in the gastrocnemius muscle after 4 weeks of exercise training were analyzed using Western blotting. CON, control; RL, low‐intensity running; RH, high‐intensity running. Data are presented as means + SD. *p < 0.05 versus the CON group, **p < 0.01 versus the CON group

We evaluated the effect of high‐ or low‐intensity running on proteins related to metabolism and mitochondrial biogenesis in skeletal muscle. The phosphorylation level of AMPK, which is determined to play an important role in the upregulation of oxidative metabolism and gene expression related to mitochondrial function (Fan & Evans, 2017), was significantly increased in the RH group compared with the Con group (Figure 4b). The protein expression level of AMPK was significantly increased by four‐weeks of running regardless of exercise intensity (Figure 4c). The phosphorylation level of ACC, which is one of the downstream substrates of AMPK and related in promotion of fatty acid oxidation, was significantly increased in the RH group relative to the Con group (Figure 4d). The protein expression level of ACC was noted to be higher in the training groups, but no significant differences were observed between all groups (Figure 4e). The protein expression level of peroxisome proliferator‐activated receptor gamma coactivator 1‐alpha (PGC‐1α), a master regulator of mitochondrial biogenesis, was not significantly different for all groups (Figure 4f). Among the mitochondrial OXPHOS proteins, as an indicator of mitochondrial content, the protein expression level of NDUFB8 (complex I) was significantly higher in the RL group than in the CON group (Figure 4g). There was no significant difference in the protein expression level of SDHB (complex II) and MTCO1 (complex IV) between the three groups (Figure 4g). The protein expression level of UQCRC2 (complex III) was higher in the RH group than the CON group (Figure 4g). Comparatively, the protein expression level of ATP5A (complex V) was higher in the RL group than the CON group, while it was significantly higher in the RH group than the CON group (Figure 4g).

### 3.4 Mitochondria and thermogenesis in iWAT

**FIGURE 5:**
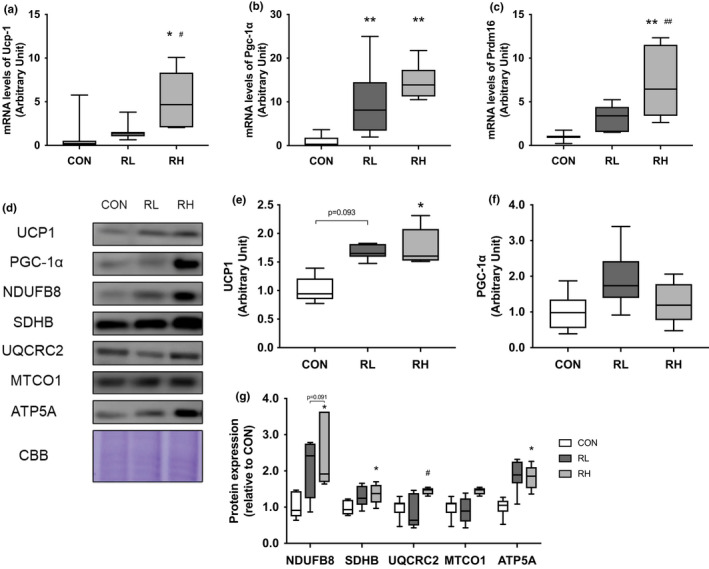
Effects of high‐ or low‐intensity running on the expression of thermogenesis markers and proteins involved in mitochondrial oxidative phosphorylation in iWAT. Gene expressions of Ucp1 (A), Pgc‐1α (B), and Prdm16 (C) in the iWAT after 4 weeks of exercise training were analyzed using RT‐PCR. Representative immunoblots are shown in (D). Protein expressions of UCP1 (E), PGC‐1α (F), and OXPHOS (G) in the iWAT after 4 weeks of exercise training were analyzed using Western blot. CON, control; RL, low‐intensity running; RH, high‐intensity running. Data are presented as means + SD. *p < 0.05 versus the CON group, **p < 0.01 versus the CON group, ^#^ *p* < 0.05 versus the RL group, ^##^ *p* < 0.01 versus the RL group

To evaluate the effect of high or low intensity running on WAT browning, we next investigated the alteration of expression level of mRNA or proteins involved in thermogenesis capacity, mitochondrial biogenesis, and OXPHOS in iWAT. The gene expression level of *Ucp*‐*1*, a key factor of thermogenesis, was significantly higher in the RH group than in the CON and RL group (Figure 5a). The gene expression level of *Pgc*‐*1* was significantly increased by four‐weeks of running regardless of exercise intensity (Figure 5b). The gene expression level of *Prdm16*, a brown adipose determination factor (Seale et al., 2011), was significantly higher in the RH group than in the CON and RL group (Figure 5c). The protein expression level of UCP1 was higher in the RL group than the CON group while significantly higher in the RH group than the CON group (Figure 5e). In contrast, no significant difference was observed between the three groups for PGC‐1α protein expression (Figure 5f). The protein expression level of NDUFB8 (complex I) was significantly higher in the RH group than the CON and RL groups (Figure 5g), while the SDHB (complex II) protein expression levels were significantly higher in the RH group than only the CON group (Figure 5g). There was no significant difference in the protein expression level of UQCRC2 (complex III) and MTCO1 (complex IV) for all groups (Figure 5g). Lastly, the protein expression level of ATP5A (complex V) was higher in the RH group than the CON group (Figure 5g).

### 3.5 Adipose tissue browning‐related proteins in skeletal muscle

**FIGURE 6:**
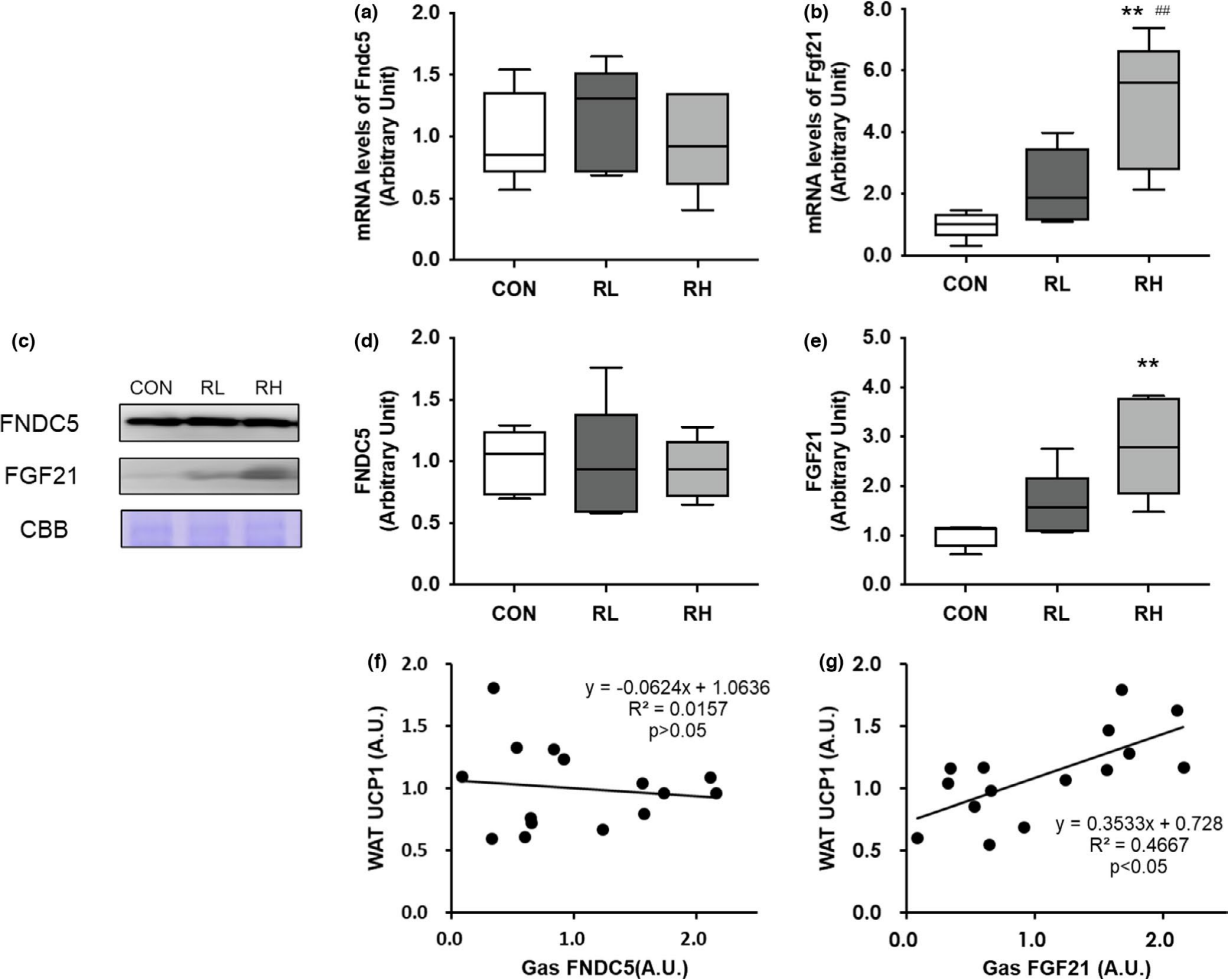
Effects of high‐ or low‐intensity running on the expression of FGF21 and FNDC5 in the gastrocnemius muscle. Gene expressions of Fndc5 (A), Fgf21 (B) in the iWAT after 4 weeks of exercise training were analyzed using RT‐PCR. Representative immunoblots are shown in (C). Expressions of FNDC5 (D) or FGF21 (E) in the gastrocnemius muscle after 4 weeks of training were analyzed using Western blot. The correlation between protein expression of UCP1 in iWAT and the protein expression of FNDC5 (F) or FGF21 (G) in gastrocnemius muscle is displayed. CON, control; RL, low‐intensity running; RH, high‐intensity running. Data are presented as means + SD. **p < 0.01 versus the CON group, ^##^ *p* < 0.01 versus the RL group

We investigated the effects of high‐ and low‐intensity running on the expression levels of FGF21 and FNDC5 in skeletal muscle. The gene expression level of *Fndc5* did not differ significantly between all groups. (Figure 6a). In contrast, the gene expression level of *Fgf21* was significantly higher in the RH group than the CON and RL group (Figure 6b). The protein expression level of FNDC5 did not differ significantly between the three groups. (Figure 6d). The protein expression level of FGF21 was significantly higher in the RH group than the CON group in gastrocnemius muscle (Figure 6b). We then examined the relationship between the degree of iWAT browning level and skeletal muscle protein, FGF21 and FNDC5. The positive correlation between the protein expression of UCP1 in iWAT and the protein expression of FGF21 in gastrocnemius muscle has been presented (Figure 6g). In contrast to FGF21, there was no significant correlation between the protein expression level of FNDC5 and the browning level of iWAT (Figure 6f). These data show that the protein expression of FGF21 in skeletal muscle might be involved with adipose tissue browning, with a dependence on exercise intensity.

## 4 DISCUSSION

In this study, we hypothesized that high‐intensity exercise can be more effective for browning of WAT in non‐obese mice compared to low‐intensity exercise. We performed high‐ and low‐intensity treadmill running on mice for four weeks. As per our findings, it was determined that four weeks of exercise did not reduce adipose tissue wet weight but did reduce adipocyte size and activated thermogenesis markers in iWAT. Furthermore, we found that high‐intensity running induced the expression of FGF21 in skeletal muscle. These results suggest that running exercise increases skeletal muscle FGF21 expression in an intensity‐dependent manner, which may be involved in adipose tissue browning.

First, we examined the effects of exercise intensity on body mass and wet weight of gastrocnemius muscle and iWAT. We found that high‐ and low‐intensity running did not affect any of these parameters. However, the change in BW was the lowest in the high‐intensity running compared to the other groups. Previous reports have shown that higher‐intensity running exercise better reduce BW gain than moderate‐intensity running exercise in obese mice (Wang et al., 2017). As we obtained similar results, this study shows that higher‐intensity exercise is more effective in suppressing BW gain even in non‐obese mice. In addition, four weeks of running significantly decreased iWAT adipocyte size. Previous studies have shown that smaller adipocytes have a higher glucose uptake and oxidative capacity (Craig et al., 1981). Moreover, other studies have reported that eight weeks of running decreases WAT weight and size (Kim et al., 2020). In this study, there was a similar decrease in iWAT adipocyte size and an increase in iWAT oxidative capacity, which proves the training effect. In contrast, the experimental period may have been too short for the adaptation of iWAT weight loss.

Next, we investigated the effects of high‐ or low‐intensity running on the level of signaling molecules for metabolism and mitochondria in gastrocnemius muscle and iWAT. In this study, high intensity running significantly increased the phosphorylation of AMPK in gastrocnemius muscle. In a previous study, the phosphorylation level of AMPK was dependent on exercise intensity (Egan et al., 2010). Thus, the exercise intensity of the RL group may have been insufficient to cause a significant increase in the phosphorylation of AMPK at the sampling time. In contrast, four weeks of running significantly increased the expression level of AMPK in gastrocnemius muscle. In a previous study, running on a treadmill (20 m/min, 30 min) for four weeks promoted the expression of the AMPK protein in skeletal muscle (Suwa et al., 2008; Takahashi et al., 2020). As previously discussed, we observed increased AMPK in the skeletal muscle of exercised mice relative to the CON group. In this study, four weeks of exercise induced metabolic adaptation of skeletal muscle. Moreover, we evaluated one of the downstream substrates of AMPK and related in promotion of fatty acid oxidation. In this study, the phosphorylation level of ACC was generally similar to that of AMPK. Thus, AMPK signaling in gastrocnemius muscle were activated depend on exercise intensity. To elucidate the mitochondrial biogenesis, we investigated the protein expression level of PGC‐1α in gastrocnemius muscle and iWAT. The protein expression of PGC‐1α in each tissue was increased by exercise, but there was no significant change. This data did not match previous reports, which showed that 8 weeks of running on a treadmill (24 m/min~, 60 min, 5 times/week) significantly increased the protein expression level of PGC‐1α in soleus muscle and iWAT in rat (Wu et al., 2014). Here, we believe that the frequency, intensity, and duration of exercise training were insufficient to cause significant alterations to of the protein expression level of PGC‐1α in each tissue.

To elucidate the mitochondrial content in gastrocnemius muscle and iWAT, we investigated the expression of OXPHOS proteins. In gastrocnemius muscle, NDFUB8 was stimulated in the RL group, but in iWAT, none of complexes changed significantly in the RL group. However, three complexes were significantly increased in RH group in iWAT. In short, mitochondrial contents of skeletal muscle may be increased by both intensities of exercise and mitochondrial contents of iWAT may be increased by higher intensity exercise.

To evaluate the metabolic adaptation of iWAT by each exercise training, we analyzed the gene expression levels of transcription factors and *Ucp1*. In this study, the gene expression of *Prdm16* in iWAT increased depend on exercise intensity. Previous study reported *Prdm16* is described as transcriptional regulators of the adipocyte thermogenic program (Seale et al., 2011; Wu, Boström, et al., 2012). Thus, the gene expression of *Ucp1* was higher in the RH group as well as in that of *Prdm16*. Additionally, we found that the protein expression level of UCP1 in iWAT was increased, depending on the exercise intensity. These results demonstrate that high‐intensity exercise may be effective for increasing mitochondrial contents and heat production capacity in iWAT. This finding suggests that we need to consider the method of exercise according to its purpose, as the alterations caused by the exercise method are different for each tissue.

Secretory factors from skeletal muscle such as FGF21, FNDC5/irisin, IL‐6, lactate, etc. are involved in the mechanism of WAT browning caused by exercise (Bostrom et al., 2012; Carrière et al., 2020; Lee et al., 2014). Of these factors, we focused on FGF21 and FNDC5. In this report, we observed significant differences in the gene and protein expression of FGF21 in the RH group relative to the CON group, but that of FNDC5 did not change. Moreover, the positive correlation between the protein expression of UCP1 in iWAT and the protein expression of FGF21 in gastrocnemius muscle is displayed in this study. However, the causal relationship between the protein expression of UCP1 in iWAT and the protein expression of FGF21 in skeletal muscle is not demonstrated in this study. It remains unclear whether our exercise protocol induced the increase of blood FGF21 concentration. Previous reports have shown that acute swimming‐based exercise increased the protein expression level of FNDC5 and blood irisin concentration in skeletal muscle, which affected adipose tissue browning (Cho et al., 2021). As FGF21 has been reported to be one of the myokines that directly induce browning of WAT as well as FNDC5/irisin (Adams et al., 2012), we believed that the same response would be observed in this study. In short, FGF21 as a myokine may be involved in one of the mechanisms of adipose tissue browning dependent on exercise intensity. Conversely, we cannot confirm the association between FNDC5 and the browning of WAT in this study. Although, FNDC5 is known as a precursor of irisin, our results suggest that irisin does not play a role in exercise intensity‐dependent browning of WAT.

There are some limitations of this study. First, we did not house the mice at thermoneutrality for mice. Recently some groups reported that mice housing at thermoneutrality led to an attenuation of some of the effects of exercise on iWAT (McKie et al., 2019; Raun et al., 2020). Thus, in terms of translatability to human, we must examine our protocol could replicated at thermoneutrality for mice in the future. Second, we did not measure circulating FGF21 and irisin concentrations in blood of mice. Although it is a significant shortcoming of our data, previous studies reported that the increase of blood FGF21 and irisin concentrations by exercise are accompanied by the increase of FGF21 and FNDC5 contents of skeletal muscle (Cho et al., 2021; Tanimura et al., 2016). Therefore, we guessed that the increase of FGF21 contents of skeletal muscle is involved in the browning of WAT.

In conclusion, we suggest that four weeks of exercise did not reduce iWAT wet weight but increased the number of smaller iWAT adipocytes. Moreover, we found higher intensity exercise was more effective for the browning of WAT. Thus, high‐intensity exercise is an appropriate exercise prescription for obesity prevention. In the future, we should examine the relationship between browning of WAT and FGF21 by changing FGF21 expression through its gene manipulation or drug inhibition. In addition, we need to examine myokines response to exercise modality and the adaptation of other organs.

## COMPETING INTERESTS

The authors declare there are no competing interests.

## AUTHOR CONTRIBUTIONS

All authors conceived and designed the project; R.T., L.K., and T.S. performed the experiments; R.T.1, L.K., and T.S. analyzed the data; R.T. wrote the paper; and R.T. and T.T. made manuscript revisions. All authors read and approved the final manuscript.

## ETHICS APPROVAL

All experimental procedures performed in this study were approved by the Institutional Animal Experiment Committee of the University of Tsukuba (20‐407).
